# Brain Tumors and Beyond: Multi-Compartment Microbiome and Mycobiome Analysis

**DOI:** 10.3390/ijms26030991

**Published:** 2025-01-24

**Authors:** László Sipos, Péter Banczerowski, János Juhász, Imre Fedorcsák, György Berényi, Nóra Makra, Zsuzsanna A. Dunai, Dóra Szabó, Loránd Erőss

**Affiliations:** 1 Department of Neurosurgery and Neurointervention, Semmelweis University, 1085 Budapest, Hungary; sipos.laszlo.kornel@semmelweis.hu (L.S.);; 2 Institute of Medical Microbiology, Semmelweis University, 1089 Budapest, Hungary; 3 Faculty of Information Technology and Bionics, Pázmány Péter Catholic University, 1083 Budapest, Hungary; 4 HUN-REN-SU Human Microbiota Research Group, 1052 Budapest, Hungary

**Keywords:** brain tumor, microbiota, microbiome, mycobiota, mycobiome, gut-brain-axis, tumor microenvironment, bacteria, fungi

## Abstract

Brain tumors are frequently diagnosed diseases in which etiology and progression largely depend on mutations and genetic factors. Additionally, recent reports document that the microbiome may influence tumor growth, tumor microenvironment, and response to therapy. Our goal was to examine the extent to which the bacterial composition—microbiota—and fungal composition—mycobiota—characteristic of the tumor and its microenvironment correlate with the composition of the gut and blood microbiota and mycobiota in five randomly selected brain tumor patients. The bacterial composition of the tumor, tumor-adjacent tissue (TAT), blood, and gut samples of the five patients were analyzed by 16S rRNA and ITS-based sequencing in order to determine the bacterial and fungal composition. The gut microbiome and mycobiome composition showed individual and tissue-specific signatures in each patient. The microbiome composition of the blood, TAT, and tumor tissue was very similar in each patient, dominated by *Klebsiella*, *Enterococcus*, *Blautia*, and *Lactobacillus* spp. In contrast, the mycobiome composition of the blood, TAT, and tumor showed a diverse, individual picture. The most common fungal species in the blood and TAT were *Tomentella*, *Didymosphaeria*, *Alternaria*, *Penicillium*, *Mycosphaerella*, and *Discosia*. The blood and TAT mycobiome were similar to each other but unique and characteristic of the patients. In contrast, in the tumor tissues, *Alternaria*, *Malassezia*, *Schizophyllum*, and *Tomentella* genus were the most common fungi genus. Our results showed that the presence of fungi in tumors shows a unique pattern that is independent of the pattern observed in the gut, blood, and tumor environment and that the effects of the mycobiome are distinct and cannot be associated with those of the microbiome. Elucidating the role of fungi in tumors and exploring the relationship between fungi and brain tumor types may open up further therapeutic options.

## 1. Introduction

Central nervous system (CNS) tumors account for a significant proportion of cancer-related deaths in adults and children. The most common types of CNS tumors are meningeomas (39.0%), pituitary tumors (17.1%), and glioblastomas (14.3%) [1,2]. Previous studies have identified various genetic alterations that may underlie the molecular basis of these diseases [3,4,5,6]. However, therapies targeting these pathways are, in general, either unsuccessful or provide minimal survival benefits [7,8].

The gut-brain axis (GBA) is the bidirectional communication network between the gastrointestinal tract and the CNS. This complex interaction involves neural, endocrine, immune, and metabolic pathways and has received significant attention in recent years, particularly for its role in neurological and psychiatric disorders. The focus of numerous current reports is how the GBA may influence brain tumors, although this is an emerging and relatively unknown area [9,10,11].

The gut microbiota influences systemic immunity, which may affect tumor progression or suppression in the brain. The immune response modulatory effect of gut bacteria is well known. On the one hand, short-chain fatty acids and other metabolites produced by gut bacteria can cross the blood-brain barrier (BBB) and influence neuroinflammation and the tumor microenvironment. On the other hand, gut dysbiosis (an imbalance of gut bacteria) leads to increased production of pro-inflammatory cytokines, which may contribute to neuroinflammation, a known factor in the progression of brain tumors. Furthermore, gut dysbiosis has been shown to cause chronic inflammation by infecting the BBB, potentially allowing tumor cells to migrate more easily or enhance their proliferation rate. Gut microbes produce neurotransmitters such as serotonin and GABA, which may indirectly influence brain tumor biology by altering the CNS environment or immune response. The connection between the gut microbiome and brain tumors is part of a concept known as the “microbiome-brain-gut axis.” The existence of this axis suggests that the gut microbiota may influence cranial nerve signaling and immune regulation in the CNS, processes that may impact tumor pathogenesis and treatment outcomes [12,13].

Studies have shown that brain tumor patients have different gut microbiome profiles compared to healthy individuals. Specifically, cancer patients harbor an increased presence of pathogenic bacteria, such as *Fusobacterium* and *Porphyromonas*, while beneficial bacteria, such as *Bifidobacterium*, are significantly reduced in the gut [12,14]. This gut dysbiosis may contribute to the inflammatory environment associated with tumor growth. The brain tumor microenvironment (TME) is a complex and dynamic ecosystem that plays a critical role in tumor development, progression, and response to treatment. New evidence suggests that bacteria in brain tumors contribute to the development of TME. These intratumoral bacteria may interact with tumor cells and components of the immune system, potentially influencing tumor behavior and therapeutic responses [12,13]. For example, certain bacterial taxa identified in metastatic brain tumors have been associated with innate immune responses, indicating an active host response to these microbes [14].

We aimed to examine the extent to which the bacterial composition—microbiota—and fungal composition—mycobiota—characteristic of the tumor and its microenvironment—tumor-adjacent tissue—correlate with the composition of the gut and blood microbiota and mycobiota in five randomly selected brain tumor patients.

## 2. Results

### 2.1. Patients Characteristics

Five patients with brain tumors were randomly selected in September 2024. The patients were admitted and underwent surgery at the Neurosurgery and Neurointervention Clinic at Semmelweis University, Budapest, Hungary, with a diagnosis of brain tumor. The patients’ diagnosis was glioblastoma (GBM), anaplastic meningeoma (AM), metastatic breast cancer (MBC), and metastatic hepatic cancer (MHC). The following abbreviations were used for the patients: P1_GBM, P2_GBM, P3_AM, P4_MBC, and P5_MHC. None of the patients received antibiotic treatment in the year before the operation. Before the neurosurgical procedure, gut and blood samples were collected from the patients prior to perioperative antibiotic administration. All patients received one dose of cephazolin as perioperative antibiotic prophylaxis.

### 2.2. Microbiota Diversities of Patient Samples

**Figure 1:**
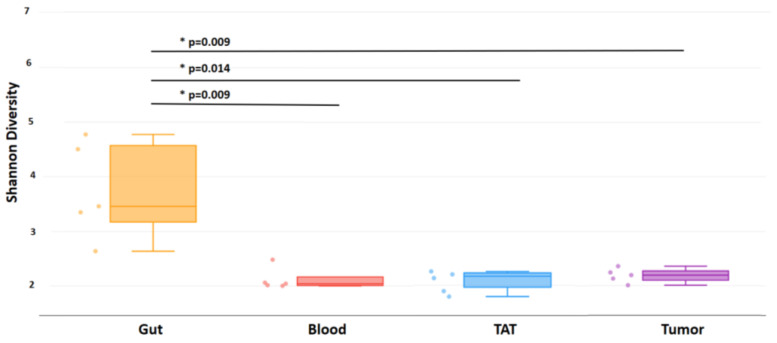
Shannon diversity of clinical samples by 16S rRNA analysis, organized by sample type. Box plots show the distribution of diversities in each group. The line shows the *p*-value between the different groups. The *p*-values of the Wilcoxon rank-sum test between groups are shown. *p*-values were considered significant when *p* < 0.05 and marked by an asterix (*).

The bacterial alpha diversity of patients’ samples—gut, blood, TAT, and tumor—was calculated using the Shannon index. The bacterial alpha diversity was the highest in the gut samples. The alpha diversity was low in blood, tumor-adjacent tissue, and tumor tissues. There was a significant difference between the gut and all the other sample types: tumor (*p* = 0.009), tumor-adjacent tissue (*p* = 0.014), and blood (*p* = 0.009) samples according to the Shannon index, shown in Figure 1. However, there was no significant difference between the alpha diversity among the other samples by Shannon indexes (Figure 1).

**Figure 2:**
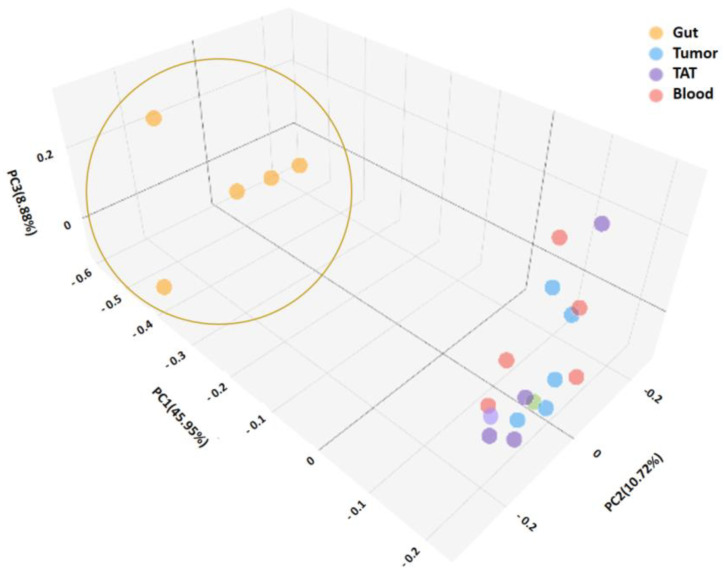
Principal coordinate analysis of microbiome by the Jaccard method. The gut samples are grouped separately and marked with a brown circle.

The beta diversity of the different samples was calculated by the Jaccard method. Based on the beta diversity, the gut samples were significantly different from other sample types, which was also confirmed by the principal component analysis shown in Figure 2.

#### 2.2.1. Consistency of the Microbiota Composition

**Figure 3:**
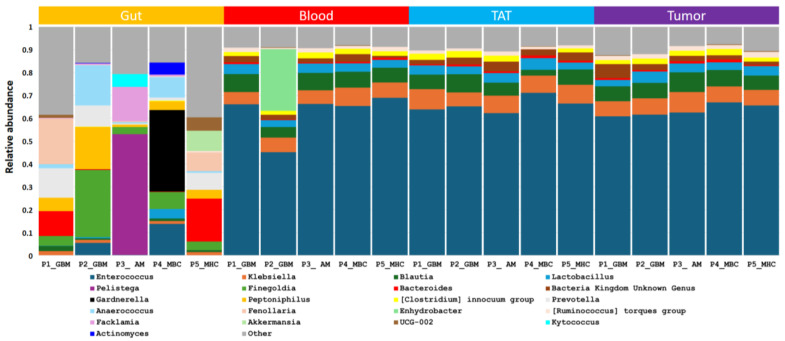
Genus level abundances of bacteria from the different patients and samples by 16S rRNA sequencing.

In tumor samples, the most abundant phyla detected taken as a whole were *Firmicutes* with an abundance of 83.79%, followed by *Proteobacteria* (12.44%) and *Bacteroidota* (0.9%). Similar ratios for the most abundant phyla were observed in tumor-adjacent tissue—*Firmicutes* (83.69%), *Proteobacteria* (12.44%), and *Bacteroidota* (0.87%)—and in blood—*Firmicutes* (80.03%), *Proteobacteria* (16.76%), and *Bacteroidota* (0.68%). In gut samples, the order was as follows: *Firmicutes* (54.63%), *Proteobacteria* (14.58%), *Bacteroidota* (13.91%), *Actinobacteria* (12.75%), *Verrucomicrobiota* (1.78%), *Campylobacterota* (1.53%), and *Fusobacteriota* (0.51%). The most abundant genera in tumor, TAT, blood, and gut samples are shown in Figure 3. The most abundant genera in tumor, tumor-adjacent tissue, and blood samples taken as a whole were *Enterococcus*, *Klebsiella*, *Blautia*, *Lactobacillus*, *Clostridium innocum* group, and *Ruminococcus*. In gut samples, the most abundant genera were *Pelistega*, *Finegoldia*, *Fenollaria*, *Facklamia*, *Prevotella*, *Kytococcus*, *Peptoniphilus*, *Escherichia-Shigella*, *Gardnerella*, *Anaerococcus*, *Dialister*, *Akkermansia*, *Campylobacter*, *Ezakiella*, *Actinomyces*, *Bacteroides*, *Blautia*, *Klebsiella*, and *Enterococcus*.

**Figure 4:**
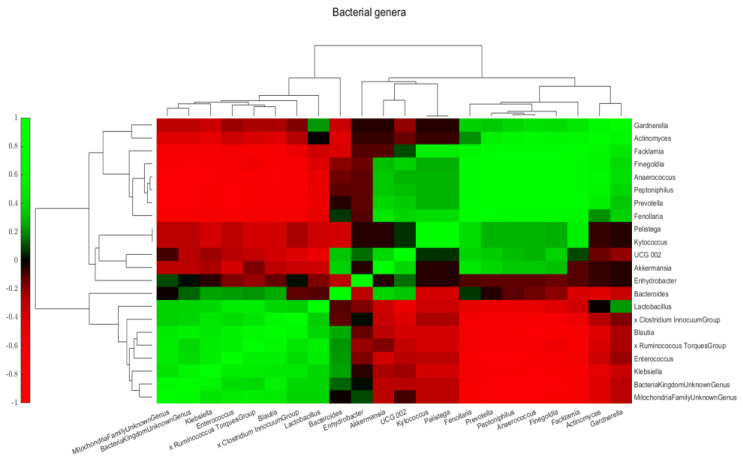
Correlation of most common (>1% on average or >5% in any sample) bacterial genera for all samples. The heatmap shows the pairwise correlation (Spearman’s rho) between selected bacterial abundances. Colors correspond to the rho values: deeper red colors represent stronger negative, and deeper green colors have stronger positive correlations. The rho values between the examined taxa are included in Supplementary Table S1.

The heatmap shows co-occurring clusters of frequent bacteria, showing an increase with some genera or an inverse relationship with others. The correlation of the most common bacterial genera in all samples is shown in Figure 4. Two bacterial groups were observed. One group included *Enterococcus* spp., *Klebsiella* spp., *Blautia* spp., *Ruminococcus* spp., *Lactobacillus*, and *Clostridium inocuum* group. The other group included *Prevotella* spp., *Gardnerella* spp., and *Actinomyces* spp.

#### 2.2.2. Mycobiota Diversity in Different Patient Samples

**Figure 5:**
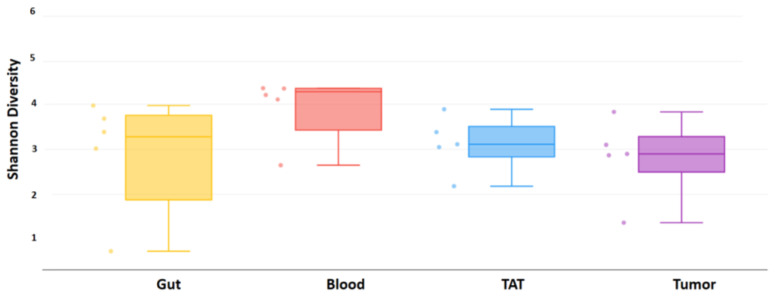
Shannon diversity of clinical samples by ITS analysis, organized by sample type. Box plots show the distribution of diversities in each group.

The fungal, mycobiome, and alpha diversity were calculated using the Shannon index. No significant difference was between the alpha diversity among any samples (Figure 5).

The beta diversity of the different samples was calculated by the Jaccard method. No significant differences were observed.

#### 2.2.3. Mycobiota Structure of Different Samples

**Figure 6:**
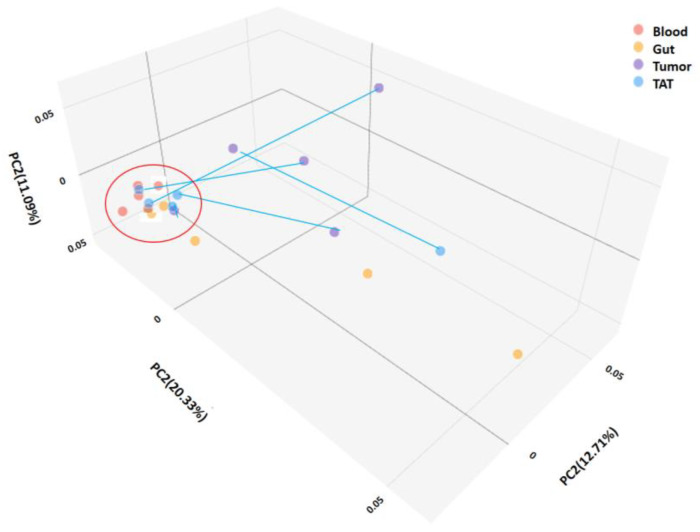
Principal component analysis of the mycobiome of the different samples. Blood samples are marked in red, gut samples in yellow, tumor samples in purple, and tumor-adjacent tissue (TAT) samples in blue. The blue lines indicate tumor and tumor-adjacent tissue samples from the same patient. The red circle indicates a group of samples that are very similar.

In contrast, mycobiome principal component analysis of different clinical samples shows that the composition of tumor and tumor-adjacent tissue samples from the same patient were generally very different. Tumor-adjacent tissue samples exhibited high similarity to blood samples. In contrast, gut samples differ from each other and other samples (Figure 6).

**Figure 7:**
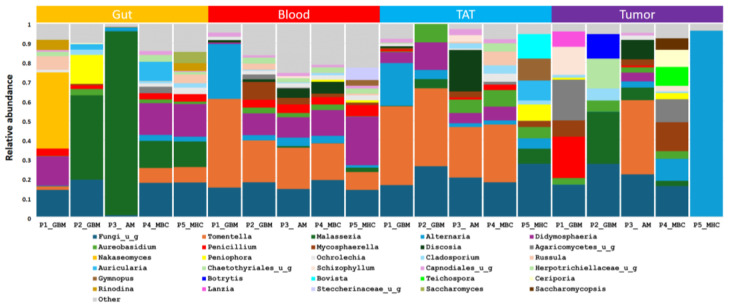
Genus level abundances of fungi from the different patients and samples by ITS sequencing.

Greater variability was found in the gut at the genus level, so characteristic features were not detected. *Malassezia* was presented in four samples (P1_GBM, P3_AM, P4_MBC, and P5_MHC) (Figure 7). The blood and TAT samples showed more similarity (Figure 6), and the most common genera were *Tomentella*, *Alternaria*, and *Didymosphaeria*. At the genus level, the tumor samples were variable; in the tumor tissues of P5_MHC, the mycotoxin-producing *Alternaria* was the only fungi; and in P4_MBC *Alternaria* and *Mycosphaerella* were the most abundant. There was no Alternaria in tumor tissue, but *Malassezia* and *Tomentella* were very common genera (Figure 7).

**Figure 8:**
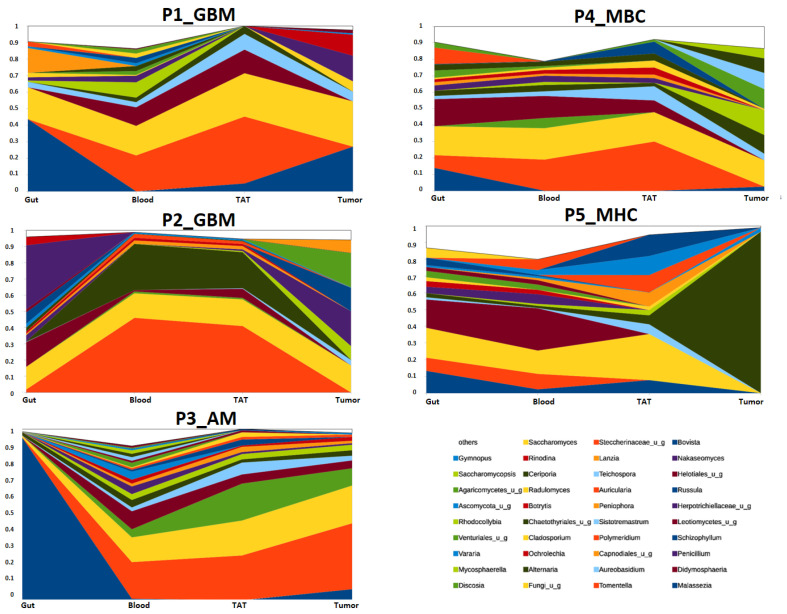
Genus level abundances of fungi from the different patients—P1_GBM, P2_GBM, P3_AM, P4_MBC, P5_MHC -and different clinical samples: gut, blood, tumor-adjacent tissue, and tumor.

Figure 8 shows the different samples connected by patients and the relative abundance changes in different fungi genera. Generally, in patients P1_GBM, P2_GBM, P3_AM, and P4_MBC, the blood and TAT abundances are similar, while P5_MHC has different patterns. The tumor fungi community in P3_AM was similar to the blood and TAT; however, in other patients, P1_GBM, P2_GBM, P4_MBC, and P5_MHC, the tumor fungi patterns were distinct from those in the TAT and blood. P4_MBC and P5_MHC have similar gut and blood compositions. P1_GBM, P2_GBM, P3_AM, and P4_MBC *Tomentella* were common in blood and TAT.

**Figure 9:**
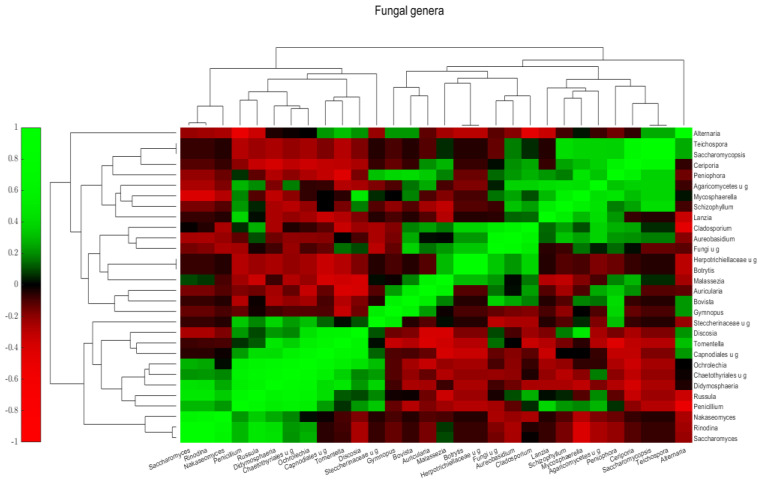
Correlation of most common (>1% on average or >5% in any sample) fungi genera for all samples. The heatmap shows the pairwise correlation (Spearman’s rho) between selected bacterial abundances. Colors correspond to the rho values: deeper red colors represent stronger negative, and deeper green colors have stronger positive correlations. The rho values between the examined taxa are included in Supplementary Table S1.

The heatmap shows co-occurring clusters of frequent fungi, showing an increase with some genera and an inverse relationship with others. The correlation of the most common fungi in all samples is shown in Figure 8. Three fungal groups were observed. One group includes *Tomentella*, *Discosia*, *Didymosphaeria*, and *Penicillum*. The second group includes *Cladosporium*, *Aureobasidium*, and non-specified fungi. The third group includes *Teichospora*, *Saccharomyces*, *Ceriporia*, and *Schizophyllum*. *Alternaria* and *Malessezia* do not fit into these categories, although *Alternaria* has a positive correlation with *Discosia* and *Tomentella* (Figure 9).

### 2.3. Intra- and Interkingdom Correlation Among the Most Abundant Bacterial and Fungal Genera

We examined bacterial and fungal interactions in order to demonstrate positive and negative interactions. Given that the gut sample showed significant differences in terms of bacterial composition, we first looked at the bacterial-fungal interactions in all samples, and then the intestinal samples were removed.

**Figure 10:**
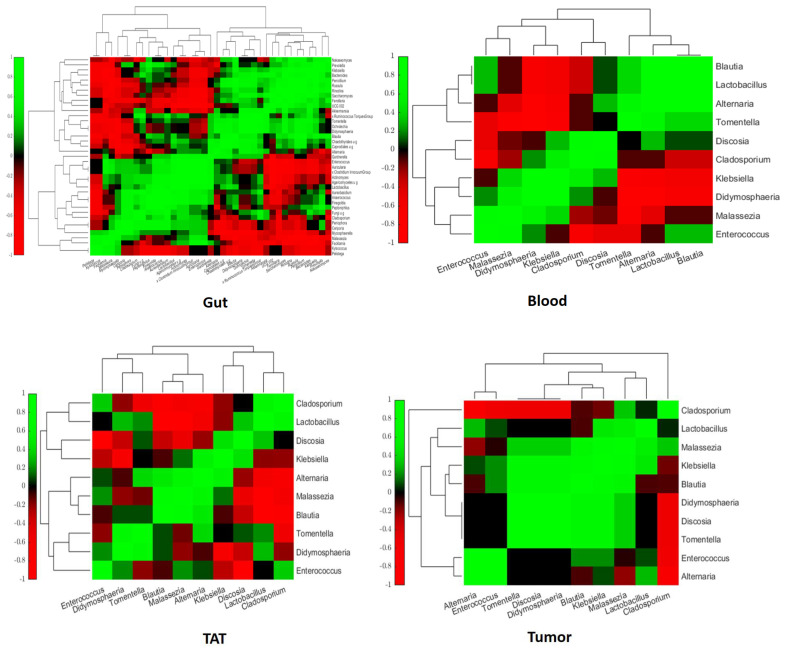
Spearman correlation coefficients (rho) between the selected bacterial and fungal genera are the values in the heat maps from blood, from TAT and from tumor tissues. The rho values between the examined taxa are included in Supplementary Table S1.

We tried to establish bacterial and fungal interactions in the blood where *Blautia*, *Lactobacillus*, *Alternaria*, and *Tomentella* positively correlated with each other, as did *Discosia*, *Cladosporium*, and *Klebsiella*. *Klebsiella*, *Didymosphaeria*, and *Malassezia* positively correlated with each other (Figure 10). *Enterococcus* positively correlated with *Malassezia*, *Lactobacillus*, and *Blautia*, as did *Alternaria* and *Discosia*. However, *Cladosporium* negatively correlated with *Lactobacillus* and *Blautia. Malassezia* negatively correlated with *Tomentella* and *Alternaria. Enterococcus* negatively correlated with *Cladosporium*, *Discosia*, and *Tomentella. Klebsiella* and *Didymosphaeria* negatively correlated with *Tomentella*, *Alternaria*, *Lactobacillus*, and *Blautia*.

Investigating the interaction in TAT, *Cladosporium* positively correlated with *Lactobacillus* and *Enterococcus*. *Lactobacillus* positively correlated with *Discosia* and *Didymosphaeria*. *Klebsiella* positively correlated with *Discosia* and *Alternaria* (Figure 10)*. Alternaria* positively correlated with *Tomentella*. *Malassezia*, *Alternaria*, and *Blautia* positively correlated with each other. *Tomentella* positively correlated with *Didymosphaeria. Enterococcus* positively correlated with *Malassezia* and *Didymosphaeria*. However, *Didymosphaeria* negatively correlated with *Enterococcus. Alternaria*, *Malassezia*, and *Blautia* negatively correlated with *Discosia*, *Lactobacillus*, and *Cladosporium. Malassezia* and *Cladosporium* negatively correlated with *Tomentella* and *Didymosphaeria. Klebsiella* negatively correlated with *Lactobacillus* and *Cladosporium*.

In tumor tissue, *Cladosporium* positively correlated with *Malassezia. Lactobacillus* positively correlated with *Alternaria*. *Alternaria* and *Enterococcus* positively correlated with each other, as did *Malassezia*, *Lactobacillus*, and *Klebsiella*. *Malassezia*, *Klebsiella*, *Blautia*, *Didymosphaeria*, *Discosia*, and *Tomentella* positively correlated with each other (Figure 10). However, *Malassezia* negatively correlated with *Alternaria*. *Cladosporium* negatively correlated with *Alternaria*, *Enterococcus*, *Tomentella*, *Discosia*, and *Didymosphaeria*.

## 3. Discussion

Our present study aimed to assess the extent to which the gut, blood, tumor, and tumor-adjacent tissue microbiomes are similar and correlate with each other in brain tumors with regard to the brain-gut axis. Recent reports about the gut microbiome in various diseases and tumors primarily concern the bacterial composition and neglect the mycobiome composition. The fungal composition of gut flora is influenced by several factors such as age, host genetics, host immunity, diet, and medication [15,16], as well as the bacterial microbiome that impacts the mycobiome through inter-kingdom interactions [16]. The composition of the gut microbiome significantly influences the development of tumors and the effectiveness of anti-cancer treatments such as chemotherapy, radiotherapy, and checkpoint inhibitors [9,10,11]. Analysis of the mycobiome in cancer patients has shown a pattern of alteration common among studies. For example, fecal mycobiota analysis indicates a significant increase in the *Ascomycota: Basidiomycota* ratio in colorectal cancer and polyp patients compared with controls [17].

Although the concept of the blood microbiome in healthy individuals is still debated, disruption of mucosal integrity in various diseases may exacerbate microbial translocation into the blood. In contrast to the healthy gut, which is known to be characterized by the phyla *Firmicutes* and *Bacteroidetes*, the healthy blood microbiome is apparently dominated by *Proteobacteria*, *Actinobacteria*, *Firmicutes*, and *Bacteroidetes*. In fact, some specific blood compounds have been localized where the healthy blood microbiome is found, including in the buffy coat, within peripheral blood mononuclear cells, such as commensal bacteria associated with red blood cells and in their extracellular vesicles [18]. The composition of the blood microbiome has been studied in various diseases, such as cardiovascular diseases, immunological diseases, liver and respiratory diseases, skin diseases, and cancer [19]. The preliminary results suggest that the blood microbiome could be associated with different tumors but not with their stage. However, further confirmation is needed in the future. In pancreatic cancer, significant microbial heterogeneity in the tumors and bloodstream distinguishes the different cancer types [20]. Furthermore, in gastric cancer [21], myeloid malignancies [22], colorectal cancer [23], non-small cell lung cancer [24], and distinct blood microbiome profiles are also demonstrated.

As opposed, the composition of the blood microbiome in primary central nervous system solid tumors is unknown and has not been investigated so far. To our knowledge, this is the first report on the composition of the blood microbiome in primary brain tumors, glioblastoma, and anaplastic meningioma.

Here, we examined only five patients, and two of them had solid tumor metastasis. One patient had breast cancer metastasis, and her blood microbiome profile -*Enterococcus* spp., *Klebsiella* spp., *Blautia* spp., and *Lactobacillus* spp.- was different from the microbiome profile of breast cancer patients observed in a cross-sectional study described by An et al. The occurrence of the genera *Citrobacter*, *Bacteriodes*, *Enterobacter*, and *Bifidobacterium* was more frequent in the blood of breast cancer patients, while a lower abundance of the genera *Staphylococcus*, *Lactobacillus Fusobacterium*, *Porphyromonas*, and *Actinomyces* was observed in the bloodstream [25]. Furthermore, in a cross-sectional study of hepatocellular carcinoma genera, *Staphylococcus*, *Acinetobacter*, *Klebsiella*, and *Trabulsiella* were increased while *Pseudomonas*, *Streptococcus*, and *Bifidobacterium* were reduced [26]. In our other patient with a brain metastasis of hepatocellular carcinoma, *Enterococcus* spp., *Klebsiella* spp., and *Blautia* spp. were the dominant genera.

Based on our preliminary results, the blood microbiome profile of the three primary brain tumor patients showed a very similar picture, with *Enterococcus* spp. dominating, followed by *Klebsiella* spp., *Blautia* spp., and *Lactobacillus* spp. being the most common. Given that these bacteria are characteristic of the intestinal flora, we assume that they entered the blood as a result of damage to the intestinal epithelial integrity.

Given that similar blood microbiome profiles can be observed in both primary and metastatic brain tumors, we hypothesize that the blood microbiome profile is specific to the presence of the brain tumor and not to its type. The intestinal and tumor microbiome influences BBB permeability, and in the absence of normal intestinal flora, BBB permeability is significantly higher, which is the result of reduced expression of the tight junction proteins occludin and claudin-5 [27]. We assume that there is a relationship between intestinal epithelial integrity and blood-brain-barrier integrity and that blood, tumor, and tissue samples.

The mycobiome has been studied from many aspects, such as the intestinal mycobiome, lung mycobiome, skin mycobiome, etc. [28]. However, no one has studied blood mycobiome, especially the bloodstream mycobiome of cancer patients, and particularly not the blood mycobiome of central nervous system cancer patients. Despite the low patient number, the significance of this study is that, in contrast to the similar blood microbiome profiles of patients with central nervous system tumors, the blood mycobiome of patients shows a different and unique individual pattern. Studies conducted on a larger number of patients may demonstrate in the future whether the blood mycobiome has a predictive value regarding the type of cancer, its stage, the effectiveness of treatment, and the prognosis of the disease.

The presence of bacteria in tumor tissue has recently come into focus. A comprehensive analysis of the tumor microbiome in seven cancer types—including breast, lung, ovarian, pancreatic, melanoma, bone, and brain—found distinct microbiome composition for each tumor type and observed a particularly rich and diverse microbiome in breast cancer [29]. Bacteria were found within tumors and localized mainly intracellular in the tumor cells and immune cells. There is an association between the bacteria found in tumors and their predicted functions, tumor types and subtypes, and responses to immunotherapy [30].

Our preliminary data with low patient numbers do not establish whether intratumor bacteria or fungi play a causal role in the development of cancer or whether their presence simply reflects infections of established tumors [31,32,33]. As tumors develop, their disorganized, leaky vasculature may allow circulating bacteria and fungi to enter, and the immunosuppressed environment may provide a refuge for them [33,34]. Bacteria belonging to the *Proteobacteria* and *Firmicutes* phyla account for most of the detected bacterial sequences in intratumor tissues in all cancer types, but the *Proteobacteria* to *Firmicutes* (P/F) ratio appears to vary between tumor types [29]. Intratumor bacteria may also arise from the normal-adjacent tissue, as was confirmed earlier [30], which can explain the high similarity. We found similar trends between the tumor microbiome and its tumor-adjacent tissue microbiome. Confirming earlier results, *Firmicutes* and *Proteobacteria* phyla were presented in more than 90% of our five tumor samples and five tumor-adjacent tissues.

Many studies described the similarity of the composition of the tumor microbiota and the normal-adjacent tissue (NAT) microbiome in terms of bacterial composition, which was also clear in our case. However, for different tumors, most of the tumor mycobiome and NAT mycobiome were similar. However, in breast tumors, differences are reported regarding the tumor and NAT mycobiome [29]. The same trend was observed in our preliminary study. Although we examined clinical samples of only five brain tumor patients, in all five cases, the TAT, which in our cases corresponds to NAT, had a different mycobiome composition from the tumor mycobiome, similar to that observed in breast cancer. Another interesting aspect of our preliminary study was the newly observed high degree of similarity between TAT and blood mycobiome.

Human tumor tissues contain a variety of fungi in addition to tumor-associated bacteria [31,35]. In addition to *Saccharomycetales* [35], other fungi, including *Candida albicans*, *Malassezia globosa*, *Malassezia restricta*, and *Blastomyces gilchristii*, may also be present in various types of human cancers [31,35,36], including prostate [37], ovarian [38], and esophageal [39]. Fungi have been identified in several other tumors [31], for example, in colorectal carcinoma—*Saccharomyces*, *Candida*, and *Aspergillus*, among others [35,40,41,42]. In pancreatic tumors, *Alternaria* and *Malassezia* [43]; in breast tumors, *Blastomyces*, *Malassezia*, and *Cladosporium* [44]; in hepatocellular carcinoma, *Malassezia* and *C. albicans*.; and in oral squamous cell carcinoma, the role of *C. albicans* was confirmed [45].

In our five cases, among the fungal species observed in the tumors, *Alternaria* genera were dominating, but *Cladosporium*, *Discosium*, and *Didymosphera* were also present in larger quantities. The role of *Alternaria* in pancreatic tumors and esophageal tumors has been described [43,46]. In our current preliminary study, *Alternaria* was only detected in samples from metastatic breast and hepatocellular carcinoma. *Malassezia* has also been described in known tumors, pancreatic, breast, ovarian, lung, and hepatocellular carcinomas, and *Cladosporidium* in breast and ovarian tumors [37,38,43,44,45]. The role of *Tomentella* is unknown. However, the observed differences between the tumor and TAT fungal composition may suggest a different pathomechanism for fungi. Considering that fungi are eukaryotic cells, they may be present in the tissue before cancer development. Whether or not bacteria and fungi play a causal role in tumorigenesis, it is of interest to further explore the effects that intratumor bacteria and fungi may have on different phenotypes of cancer cells and on the immune system and its interactions with tumor cells [47].

Fungal–bacterial interactions have been widely reported, often with clinical significance. Recently, these interactions have gained attention due to their impacts on human health [47]. Understanding the nature of these interactions is key for the prevention and management of polymicrobial infections and channeling them to gain potential beneficial effects. An imbalance between these microbes (also known as “dysbiosis”) may predispose the host to a variety of chronic fungal infections and diseases at local and distant sites [48]. Moreover, having a better understanding of such interactions may be helpful in the identification of novel targets for future anti-cancer treatments.

In summary, the gut-brain axis also plays an important role in brain tumors, and the role of the gut-blood-TAT-tumor pathway is very prominent. While the blood microbiome correlates with the tumor and TAT microbiome, in the case of mycobiome, the blood and TAT mycobiome are also similar but do not provide information about the tumor mycobiome. Our results confirm the role of blood microbiome and mycobiome as potential biomarkers in brain tumors. Based on our knowledge, this is the first report to investigate brain tumor patients, the blood microbiota and mycobiota, the pathway of gut-blood-TAT-tumor, and bacterial-fungal interactions. While the exact role of the microbiome and mycobiome in malignant brain tumors is still being investigated, the connection between gut health, immunity, and inflammation opens up potential new avenues for understanding brain tumor mechanisms. Further research will clarify how making designer changes in the microbiome might improve outcomes for brain tumor patients.

The limitations of this study are the small number of patients and the lack of a negative control group due to the nature of the study, which involved central nervous system tumor samples. Another shortcoming of this study is that the microbiome and mycobiome of the different samples were examined only at one point, at the time of surgical intervention, and the patients were not followed up yet. Based on these, we will increase the number of patients in the future, and the composition and alterations of the patient’s microbiome and mycobiome will be followed up, allowing monitoring of the progression of the disease and the effectiveness of various therapies.

## 4. Materials and Methods

### 4.1. Study Design

In our prospective study, five patients with brain tumors were randomly selected in September 2024. The patients underwent surgery and craniotomy at the Neurosurgery and Neurointervention Clinic at Semmelweis University, Budapest, Hungary with a diagnosis of brain tumor. The characteristics of the involved patients are shown in Table 1.

The patients included in this study did not receive antibiotic treatment in the year before surgery; however, the other medications that they were taking were as follows: P1_GBM: perindopril tert-butylamine, pantoprazole, potassium chloride, and methylprednisolone; P2_GBM: levetiracetam, dexamethasone, potassium chloride, naproxen, alprazolam, levothyroxine, bisoprolol, valsartan, allopurinol, rabeprazole, and metformin; P3_ AM: perindopril and carbamazepine; P4_MBC: letrozole, dexamethasone, potassium chloride, and furosemide; P5_MHC: tramadol, enoxaparin sodium, and amlodipin.

From each brain tumor, two smaller pieces—tumor and tumor-adjacent tissue—were processed separately according to the aseptic rules for a total of ten tested samples from five patients. Brain tumor and tumor-adjacent tissue were collected according to the neurosurgical standards [49,50]. Additional blood and gut samples were collected before the operation and processed from each patient according to the aseptic rules, as well. At least 3 mL of whole blood was collected into citrate-filled VACUETTE collection tubes (Greiner Bio-One, Stonehouse, UK) and immediately frozen at –80 °C. From the anorectal region, fecal samples representing the gut were collected with a DNA/RNA Shield SafeCollect Swab Collection Kit (Zymo Research Inc., Irvine, CA, USA). All the samples were stored at −80 °C until DNA extraction.

**Figure 11:**
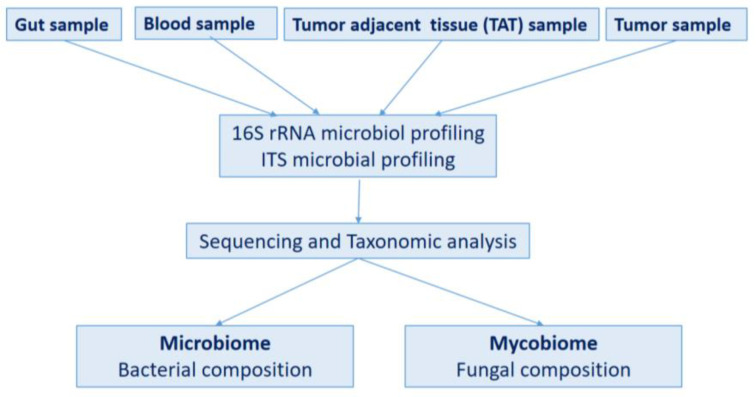
Flowchart of clinical sample processing.

The flowchart of sample processing is shown in Figure 11.

### 4.2. DNA Extraction and Characteristics

DNA isolation was performed by NucleoSpin Blood, Mini kit (Macherey-Nagel, Allentown, PA, USA) from blood samples and by ZymoBIOMICS DNA Miniprep Kit (Zymo Research Corp., Irvine, CA, USA) from the tumor, TAT and anorectal gut samples according to the manufacturer’s instructions, after enzymatic dissolution with Proteinase K (56 °C, 3 h) (Zymo Research Corp., Irvine, CA, USA). The median of isolated DNA from tumor samples was 195.2 ng/µL, from tumor-adjacent tissue 161.4 ng/µL, from blood 161.5 ng/µL and from gut samples 0.2 ng/µL.

### 4.3. 16S rRNA Microbiota Analysis

Bacterial DNA was amplified with tagged primers covering the V3-V4 region of the bacterial 16S rRNA gene. PCR and DNA purifications were performed according to Illumina’s protocol. PCR product libraries were assessed using a DNA 7500 Kit with an Agilent 2100 Bioanalyzer (Agilent Technologies, Waldbronn, Germany). Equimolar concentrations of libraries were pooled and sequenced on the Illumina MiSeq platform (Illumina, San Diego, CA, USA) using MiSeq Reagent Kit v3 (600 cycles PE) Illumina, Inc. (Berlin, Germany). All analysis procedures were performed in duplicate to avoid contamination and increase the reliability of the study. Extraction-negative controls, PCR-negative controls, and PCR-positive controls (ZymoBIOMICS Microbial Community Standard, Zymo Research Corp., Irvine, CA, USA) were included in every run to evaluate the contribution of extraneous DNA from reagents. Raw sequencing data were retrieved from the Illumina BaseSpace, and the data were analyzed by the CosmosID bioinformatics platform (CosmosID Metagenomics Cloud, app.cosmosid.com, CosmosID Inc. Rockville, MD, USA, www.cosmosid.com, accessed on 27 January 2016) described elsewhere [51].

From this starting amount, which also contains human DNA, after 16S rRNA PCR, the median DNA amount of tumor samples was 24.9 ng/µL, from tumor-adjacent tissue 25.6 ng/µL, from blood 22.3 ng/µL, and from gut samples 42.206 ng/µL. After indexing PCR, the median DNA amount of DNA from tumor samples was 10.8 ng/µL, from tumor-adjacent tissue 11.8 ng/µL, from blood 8.49 ng/µL, and from gut samples 14.8 ng/µL were amplified.

The average length of index 16S rRNA PCR products was 596 bp. From the simultaneously processed transport buffers of samples, as from negative controls, neither DNA isolation nor 16S rRNA PCR resulted in measurable amounts of DNA. A total of 1.9 million valid sequences were obtained, resulting in 1 million high-quality reads; the median number of reads within one sample was 36852.

### 4.4. ITS Mycobiota Analysis

The standard Illumina fungal metagenomic protocol was used with the following modifications to perform the ITS mycobiota analysis. In order to optimize the PCR reaction, the DNA was increased to 6.25 µL per reaction, and the primer volume was reduced to 3 µL to minimize primer dimer formation. The number of PCR cycles was increased to 30 to ensure adequate amplification of the target regions. A two-step purification was performed using 25 µL and 10 µL beads sequentially, ensuring the removal of non-specific products and primary dimers while retaining target amplicons (QuantaBio SparQ PureMag Beads). During the index PCR, step 20 cycles were to improve the yield of indexed libraries.

After ITS PCR, the median DNA amount of tumor samples was 4.61 ng/µL, from tumor-adjacent tissue 2.86 ng/µL, from blood 2.77 ng/µL, and from gut samples 1.06 ng/µL. After indexing PCR, the median DNA amount of DNA from tumor samples was 31.4 ng/µL, from tumor-adjacent tissue 58 ng/µL, from blood 23.3 ng/µL, and from gut samples 26 ng/µL amplified. The average length of index ITS PCR products was 724 bp. From the simultaneously processed transport buffers of samples, as from negative controls, neither DNA isolation nor ITS PCR resulted in measurable amounts of DNA. A total of 5.4 million valid sequences were obtained, resulting in 2.4 million high-quality reads; the median number of reads within one sample was 125,108.

### 4.5. Statistical Analysis

Statistical significance between cohorts was implemented using the Shannon index for microbiome and mycobiome alpha diversity and Jaccard for beta diversity by applying the statistical analysis support of the CosmosID bioinformatics platform. The *p*-values were calculated by the Wilcoxon rank-sum test for differences in alpha diversity (Shannon index) between groups. *p*-values were considered significant when *p* < 0.05. Principal component analysis was used for visualization by supporting the CosmosID platform [51]. Spearman correlations between bacterial and fungal taxa were calculated and visualized in MATLAB. Taxa with average relative abundance > 1% or relative abundance > 5% in any samples were shown in bar charts and correlation coefficient heatmaps.

## 5. Conclusions

The relationship between brain tumors and the microbiome and mycobiome is an emerging field of research, with growing evidence suggesting that gut and tumor-resident microbiota may influence brain tumor biology, progression, and treatment response. In addition to bacterial composition, fungal composition has been a less studied area until now. Our preliminary results showed that the presence of fungi in tumors shows a unique pattern that is independent of the pattern observed in the gut, blood, and tumor environment and that the effects of the mycobiome are distinct and cannot be associated with those of the microbiome. Elucidating the role of fungi in tumors and exploring the relationship between fungi and brain tumor types may open up further therapeutic options.

## Figures and Tables

**Table 1:** Characteristics of the selected patients with brain tumors.

Patient ID	Gender	Age	Diagnosis	Abbreviation
P1	male	48 years	Glioblastoma grade 4 (NOS)	P1_GBM
P2	female	74 years	Glioblastoma grade 4 (NOS), IDH mutant	P2_GBM
P3	male	47 years	Anaplastic meningeoma grade 3	P3_AM
P4	female	63 years	Metastatic breast cancer, ER, HR, Her2 positive	P4_MBC
P5	male	72 years	Metastatic hepatic cancer	P5_MHC

## Data Availability

The 16S and ITS datasets generated during the current study are available in the Short Read Archive (SRA) under accession numbers PRJNA1195010 and PRJNA1195037.

## Supplementary Materials

The following supporting information can be downloaded at: https://www.mdpi.com/article/10.3390/ijms26030991/s1.
